# Insights into the functional role of tomato *TM6* as a transcriptional regulator of flower development

**DOI:** 10.1093/hr/uhae019

**Published:** 2024-01-16

**Authors:** Rocío Fonseca, Carmen Capel, Ricardo Lebrón, Ana Ortiz-Atienza, Fernando J Yuste-Lisbona, Trinidad Angosto, Juan Capel, Rafael Lozano

**Affiliations:** Centro de Investigación en Agrosistemas Intensivos Mediterráneos y Biotecnología Agroalimentaria (CIAIMBITAL), Universidad de Almería, Edif. CITE II-B, Carretera de Sacramento s/n, 04120 Almería, Spain; Centro de Investigación en Agrosistemas Intensivos Mediterráneos y Biotecnología Agroalimentaria (CIAIMBITAL), Universidad de Almería, Edif. CITE II-B, Carretera de Sacramento s/n, 04120 Almería, Spain; Centro de Investigación en Agrosistemas Intensivos Mediterráneos y Biotecnología Agroalimentaria (CIAIMBITAL), Universidad de Almería, Edif. CITE II-B, Carretera de Sacramento s/n, 04120 Almería, Spain; Centro de Investigación en Agrosistemas Intensivos Mediterráneos y Biotecnología Agroalimentaria (CIAIMBITAL), Universidad de Almería, Edif. CITE II-B, Carretera de Sacramento s/n, 04120 Almería, Spain; Centro de Investigación en Agrosistemas Intensivos Mediterráneos y Biotecnología Agroalimentaria (CIAIMBITAL), Universidad de Almería, Edif. CITE II-B, Carretera de Sacramento s/n, 04120 Almería, Spain; Centro de Investigación en Agrosistemas Intensivos Mediterráneos y Biotecnología Agroalimentaria (CIAIMBITAL), Universidad de Almería, Edif. CITE II-B, Carretera de Sacramento s/n, 04120 Almería, Spain; Centro de Investigación en Agrosistemas Intensivos Mediterráneos y Biotecnología Agroalimentaria (CIAIMBITAL), Universidad de Almería, Edif. CITE II-B, Carretera de Sacramento s/n, 04120 Almería, Spain; Centro de Investigación en Agrosistemas Intensivos Mediterráneos y Biotecnología Agroalimentaria (CIAIMBITAL), Universidad de Almería, Edif. CITE II-B, Carretera de Sacramento s/n, 04120 Almería, Spain

## Abstract

Flower development is a crucial step towards the completion of the plant life cycle. Physiological processes and gene regulatory mechanisms underlying flower formation have been extensively characterized, and the implication of MADS-box transcription factors as primary regulators of flower morphology has been widely described, mainly due to the analysis of loss-of-function mutants in model species. Nevertheless, detailed characterization of allele variation in several MADS-box homologous genes from crop species remains undescribed. Here, we have characterized a tomato mutant with aberrant flower development. Mutant plants exhibit changes in petal cell identity, as well as homeotic transformations of stamens into carpelloid structures, which in most cases result in succulent organs. Molecular analysis proved that a loss-of-function mutation in the *TOMATO MADS-BOX 6* (*TM6*) gene is responsible for this mutant phenotype. Furthermore, as a result of the loss of function of *TM6*, misregulation of the transcription and mRNA processing of other MADS-box genes involved in reproductive development has been detected. Our findings demonstrate that *TM6* is a key player in the complex regulatory network of MADS-box genes controlling flower development and also provide a novel mutant that may be useful for generating male sterile lines in tomatoes.

## Introduction

With nearly 260 000 species classified into 453 families, angiosperms are undoubtedly the most successful group among terrestrial plants, mainly due to the development of a unique feature: the flower. Although there is a huge diversity of colors, forms, and morphology of flowers, the basic developmental program is highly conserved among angiosperms; indeed flowers are usually formed by four organ whorls: sepals, petals, stamens, and carpels. The organization of this structure has been the center of a large number of investigations focused on dissecting the molecular mechanisms controlling their development. The completion of these works has been the ABC model of flower development, which establishes the gene functions that control the identity of the four whorls [1].

Flower development is a physiological process controlled by a complex regulatory gene network. According to the ABC model described in the model species *Arabidopsis thaliana* L. and *Antirrhinum majus* L., the A-class gene activity specifies the development of sepals in the outermost whorl, a role assumed by the *Arabidopsis* genes *APETALA 1* (*AP1*) and *AP2* [2]. The coordinated activity of A- and B-class genes specifies petals in the second whorl. The B-class function is represented by the *Arabidopsis* genes *APETALA 3* (*AP3*) and *PISTILATA* (*PI*). Downregulation of these genes results in sepal-like petals and carpels instead of stamens in the third whorl [3]. B- and C-class genes together are responsible for stamen development, while C-class genes alone specify carpels in the fourth whorl [4]. The C-class function is represented by *AGAMOUS* (*AG*), whose loss of function causes flowers to develop petals instead of stamens in the third whorl, and another indeterminate repetition of sepals and petals is developed instead of carpels in the fourth whorl [5]. Furthermore, new gene functions have contributed to the enrichment of this model. Thus, D-class genes *SHATERPROOF 1* (*SHP1*) and *SHP2* redundantly specify ovule identity [6], whereas *SEPALLATA* (*SEP*) E-class gene function is crucial for meristem determination and the identity of all four whorls [7]. Most of ABCDE genes code for MADS-box transcription factors, a widely distributed group of proteins with many important genetic and molecular roles in the plant life cycle, that extend beyond the control of flower morphogenesis to the developmentof almost all organs, including embryo and gametophyte development [8].

The evolutionary history of ABC genes is characterized by frequent duplication events, and in some cases, there is no evidence indicating whether these duplications have led to functional divergence or to neofunctionalization of the new paralogs [9]. Despite this complex duplication background, strong evidence supports that the ABC model, as well as the basic developmental program underlying flower ontogeny, is widely conserved among angiosperms.

**Figure 1:**
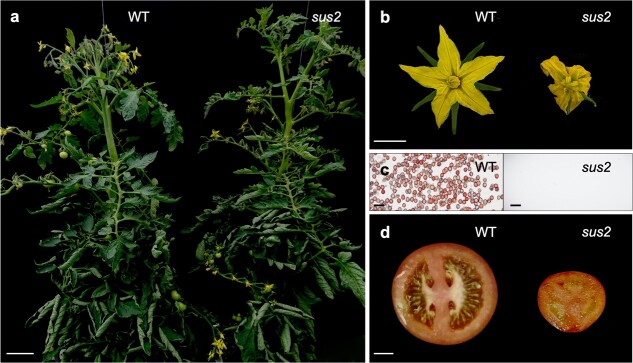
The *sus2* mutant phenotype. (a) *sus2* mutant plants show no differences in vegetative growth when compared with WT ones. (b) *sus2* flowers look smaller, and their stamens exhibit aberrant morphology. (c) Pollen viability assays performed with tetrazolium chloride proved that WT plants produce a large number of viable stained pollen, whereas in *sus2* mutant flowers no pollen grains were observed. (d) Mutant fruits are smaller and parthenocarpic (seedless). Scale bar in (a) represents 10 cm, 1 cm in (b) and (d), and 50 μm in (c)

In tomato (*Solanum lycopersicum* L.), a major vegetable crop, the genomic organization of the ABC genes has been extensively studied, although not all of their genes are supported by well-characterized mutants. Among these genes, the B-class MADS-box genes are particularly interesting as they regulate stamen development and male sterility, making them potentially valuable for hybrid seed production [10]. B-class genes in tomato are represented by the *GLOBOSA*/*PISTILLATA* (*GLO/PI*) clade, which includes the genes *TOMATO PISTILLATA* (*TPI*) and *TPIB* (syn. *SlGLO2* and *SlGLO1*, respectively), and the *DEFICIENS/APETALA 3* (*DEF/AP3*) clade, to which *STAMENLESS/TOMATO APETALA 3* (*SL/TAP3*) and *TOMATO MADS-BOX 6* (*TM6*) belong*.* All these genes are specific to the core eudicot clade and are products of major duplication events [9, 11]. Whereas *TPI*, *TPIB*, and *SL/TAP3* genes have been broadly proven to regulate petal and stamen identity, it is discussed whether *TM6* functions in a redundant manner [12, 13]. To date, *TM6* has been found to be strongly expressed in stamens and carpels of floral buds and to a lesser extent in petals [13, 14]. Functional characterization of this gene has been carried out through RNA interference (RNAi)-induced silencing [13]. However, this approach led to the conclusion that the loss of function of this gene only changes stamen morphology, while petal identity remains unaltered. Thus, functional divergence is assumed in the role of both paralogous B-class genes *TM6* and *SL/TAP3* in the control of second whorl development, as the implication of *TM6* in petal formation has not been demonstrated so far.

In this work, we have characterized a tomato male sterile mutant identified as part of an ethyl methanesulfonate (EMS) mutant collection [15], which shows clear alterations in flower morphogenesis. We have named this mutant *succulent stamens 2* (*sus2*) since most stamens in *sus2* mutant plants show a carpel-like identity and remain in mature fruits as succulent structures. Fine mapping analysis revealed that the *TM6* gene is responsible for this phenotype. The *TM6* gene belongs to a paralogous lineage of the *Arabidopsis* B-class gene *AP3* that originated as a result of a duplication event in the *AP3* lineage. The *TM6* gene was previously proposed to be implicated only in stamen development [13]. Our findings not only identify a new allele of the *TM6* gene but also shed light on its functional classification as a truly B-class gene involved in both tomato stamen and petal morphogenesis, as well as fruit development.

## Results

### *sus2* mutation impairs reproductive development

As part of the screening of an EMS mutant collection obtained in *S. lycopersicum* cv. MM, we identified an M_2_ family in which individual plants showed no vegetative development or flowering time alterations (Fig. 1a). However, some members exhibited severe abnormalities in reproductive structures. At the anthesis stage, the sepals of those mutant plants were normal, but their petals were smaller than those of wild-type (WT) plants, and their stamens remained green and appeared curled and unfused (Fig. 1b). Moreover, pollen viability assays proved that mutant stamens were unable to produce pollen, thus yielding male sterile flowers (Fig. 1c). These mutant plants produced smaller and parthenocarpic fruits, probably due to the absence of viable pollen (Fig. 1d). Genetic analysis of the mutation was performed in larger M_3_ segregating populations, where the mutant phenotype was observed in 64 of 267 plants. The chi-square statistical test confirmed that the observed segregation ratios were consistent with a monogenic recessive inheritance for the mutant phenotype (*χ*^2^ = 0.15; *P* = 0.69).

**Figure 2:**
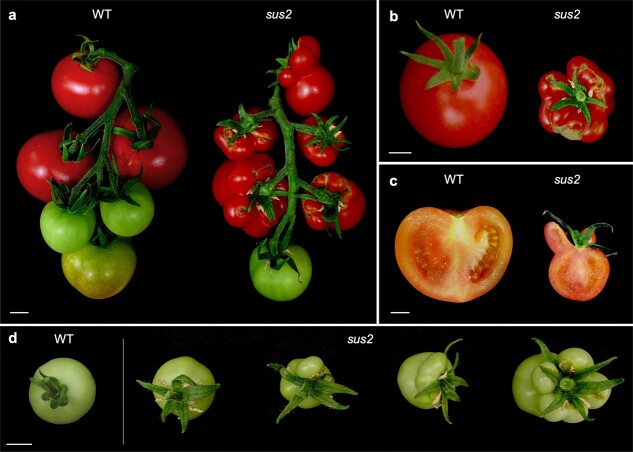
*sus2* mutant fruits develop succulent stamens. (a) All *sus2* mutant plants develop a different number of succulent stamens that fuse to carpels during fruit growth and ripening, and in the same fruit truss mutant and normal fruits can be observed. (b) An upper view of a WT and a mutant fruit where all stamens appear attached as succulent organs. (c) A longitudinal cut of a mutant fruit shows the resemblance between succulent stamens and carpel morphology. (d) Succulent stamens appear in a variable number in mutant fruits, ranging from zero to all stamens transformed into succulent structures. Scale bars represent 1 cm

Later in development, some stamens of the mutant flowers are transformed into succulent organs, which gave the mutant its name, *succulent stamens 2* (*sus2*). These transformed succulent stamens remain in the fruits during growth and ripening (Fig. 2a and b), showing a structure similar to that of carpels when observed in a longitudinal section (Fig. 2c). To characterize this aspect of the *sus2* mutant phenotype, a total of 124 M_3_ mutant plants were analyzed with the aim of determining the number of succulent stamens developed in the first 10 fruits produced by each of these plants. Succulent stamens were observed in all these mutant plants, although in varying numbers of fruits per plant. Among these fruits, 54.11% developed no succulent stamens, 33.23% developed between one and three succulent stamens, and 12.66% of the fruits developed between four and six succulent stamens (Fig. 2d). Taken together, these data indicate complete penetrance but variable expressivity in the character development of succulent stamens shown by the *sus2* mutant plants.

### *sus2* flowers display identity changes of petal and stamen primordia

**Figure 3:**
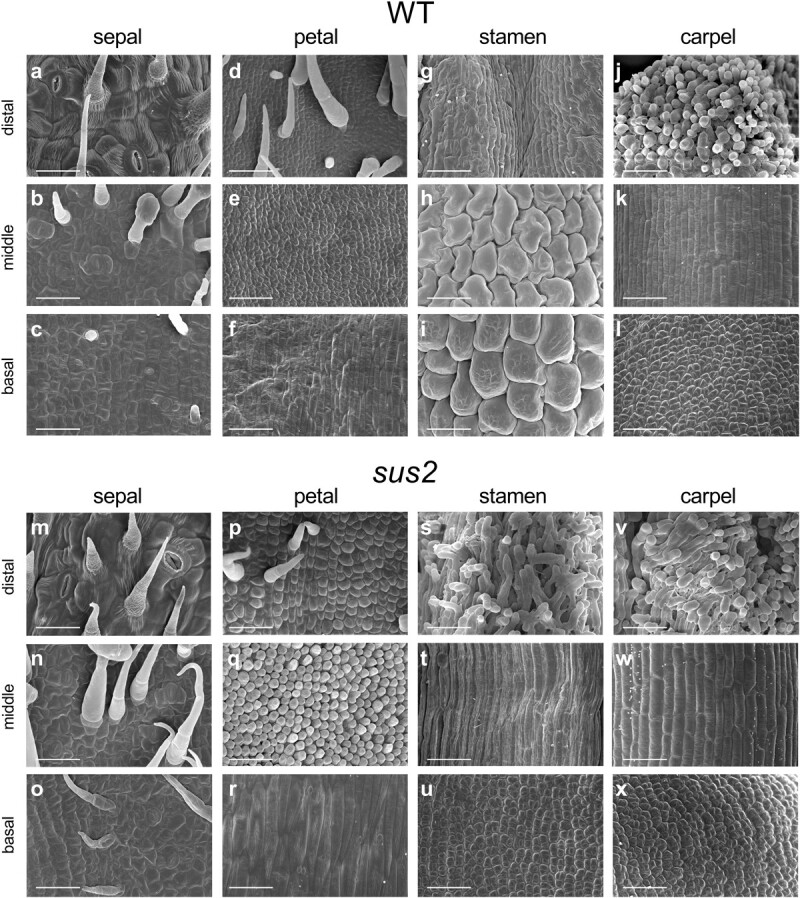
SEM analysis of distal, middle, and basal sections of anthesis flowers from WT and *sus2* mutant plants. Epidermal cell morphology in WT sepals (a–c), petals (d–f), stamens (g–i), and carpels (j–l). Epidermal cell morphology in *sus2* mutant sepals (m–o), petals (p–r), stamens (s–u), and carpels (v–x). All scale bars represent 50 μm

To elucidate the morphological changes observed in *sus2* mutant plants, we performed a scanning electron microscopy (SEM) analysis of the epidermal cell identity in the four whorls of anthesis flowers from both WT and *sus2* plants (Fig. 3). We studied the cell identity in three sections of these organs, i.e. basal, middle, and distal sections. No changes in cell identity were observed in the sepals and carpels of *sus2* plants, as the identity of the epidermal cells formed in these organs was the same as that observed in WT flowers (Fig. 3). However, this is not the case of epidermal cells of *sus2* petals, which did showed changes in cell identity, reflected in differences in size and shape when compared with epidermal cells of WT plants (Fig. 3). The identity of epidermal cells in *sus2* petals did not resemble the identity of cells in any whorl of WT plants. In addition, the morphology of epidermal cells of *sus2* stamens was quite different from that of WT ones. In the distal portion, *sus2* stamens (Fig. 3s) exhibited a papillae morphology similar to that of WT stigmas (Fig. 3j). The middle section cells of mutant stamens were elongated (Fig. 3t) and resembled those of WT styles (Fig. 3k), instead of the puzzled, rounded epidermal cells typically found in WT stamens (Fig. 3h). Finally, cells in the basal section of mutant stamen (Fig. 3u) were smaller and irregular compared with WT ones (Fig. 3i), resembling those of WT ovaries (Fig. 3l). Altogether, the homeotic changes observed in the petals and stamens of *sus2* flowers suggest that *sus2* can be considered a B-class mutant.

### Cloning and molecular characterization of *SUS2*

**Figure 4:**
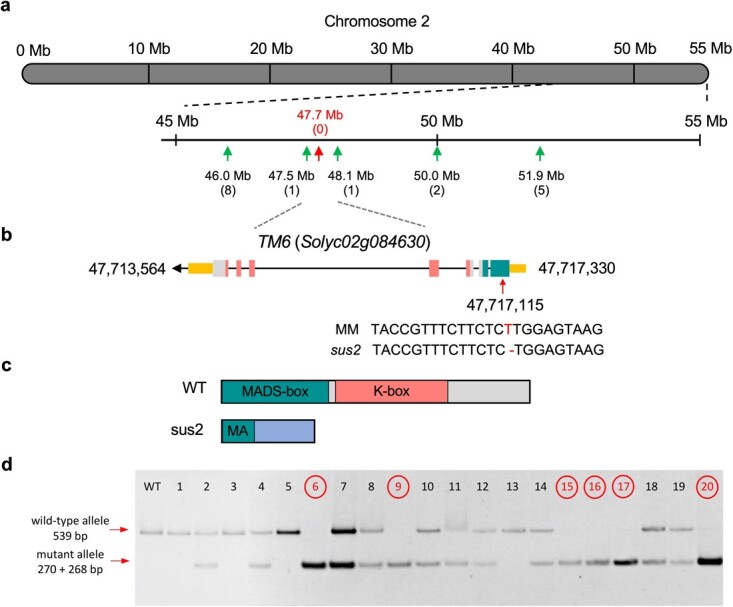
Cloning and molecular characterization of *SUS2.* (a) Mapping of an F_2_ segregating population using codominant markers identified a candidate region to harbor the *sus2* mutation on chromosome 2. Numbers in parenthesis indicate the number of recombinant chromosomes identified between the gene and each genetic marker analyzed. Note that no recombinants were found for the 47.7 Mb marker position. (b) Fine mapping of this interval was carried out by a mapping-by-sequencing approach, and variant analysis identified a single-base deletion in the first exon of the *Solyc02g084630* gene, previously named as *TM6.* Exons and introns are depicted as boxes and lines, respectively. Yellow boxes represent the 5′ and 3′ untranslated regions, while green, pink, and grey boxes represent exons coding for MADS-box, K-box and non-motif regions of the SUS2 protein respectively. (c) As a result of the *sus2* mutation the TM6 mutant protein lacks all functional domains, including most of the MADS-box domain. (d) Co-segregation test performed in 20 plants of the M_2_ segregating population for the *sus2* mutation. Plants displaying a mutant phenotype (circled) were found to be homozygous for the mutation identified in the *TM6* (*Solyc02g084630*) gene. This mutation introduces a restriction site for the *Bpm*I endonuclease, which digests the PCR amplicon from the mutant allele into 270 and 268 bp fragments. Plants showing WT phenotype were found to be heterozygous or homozygous for the WT allele, which is not digested by the *Bpm*I endonuclease

With the aim to identify the gene that underlies the *sus2* mutation, mapping strategies were carried out. An F_2_ segregating population was generated from the cross of a *sus2* mutant plant with a plant from the wild relative *Solanum pimpinellifolium* accession LA1589. A population formed by 129 F_2_ segregating plants was phenotypically evaluated. Among these, 27 *sus2* mutant plants were detected, which confirmed the monogenic recessive inheritance (*χ*^2^ = 1.14; *P* = 0.28) of the *sus2* mutant phenotype in this interspecific segregating population. Mapping was performed by genotyping all F_2_ plants with codominant markers distributed along the genome [16]. This strategy allowed us to identify a genomic region of 600 kb as a candidate for harboring the mutation located on chromosome 2 (Fig. 4a). Fine mapping was completed by a whole genome sequencing approach. We sequenced WT and mutant pools formed by equimolar amounts of DNA from 25 WT and 19 mutant F_2_ plants, respectively. Analysis of allele frequencies confirmed the location of the *sus2* mutation on chromosome 2 (Supplementary Fig. S1). Afterwards, variant analysis of the interval encompassing the candidate region allowed us to identify a unique mutation, a single base deletion in the first exon of *Solyc02g084630* gene, syn. *TM6* (Fig. 4b). The mutation detected in *sus2* causes a frameshift in *TM6*, and as a result mutant protein lacks all functional domains, including the MADS domain (Fig. 4c; Supplementary Fig. S2).

To support the causal relationship between the mutation identified by mapping-by-sequencing and the *sus2* mutant phenotype, a co-segregation analysis was carried out by using a CAPS codominant marker designed to detect the deletion identified in the *TM6* genomic sequence. The co-segregation test was performed in both M_2_ and F_2_ segregating populations (Fig. 4d), which showed that all 91 *sus2* mutant plants were homozygous for the single thymine deletion, whereas 207 and 98 phenotypically WT plants were hemizygous or lacked the deletion, respectively, indicating that the *sus2* phenotype co-segregated with the single base deletion at the *Solyc02g084630* gene. These results, combined with the absence of additional mutations in the chromosome 2 candidate region and the fact that *sus2* is a B-class homeotic mutant, have led us to conclude that the identified mutation in *TM6*, a paralogous of the Arabidopsis B-class gene *AP3*, is responsible for the *sus2* mutant phenotype.

### Transcriptomic changes in *sus2* flowers

With the aim to determine the transcriptomic changes caused by the loss of function of *TM6* in the genetic networks that control flower development, an RNA-seq analysis was carried out in floral buds of both WT and *sus2* mutant plants. Developmental stages analyzed included flowers during floral organ primordia formation i.e. FB0 (flower bud 0), FB1 (flower bud 1), and FB2 (flower bud 2), as well as flowers at pre-anthesis (PA) stage, flowers at anthesis day (AD) stage, and flowers 2 days past anthesis (AD2), as it has been described that *TM6* transcripts are more abundant in mature flower organs [13, 14]. A total of three biological replicates per genotype and developmental stage were analyzed.

**Figure 5:**
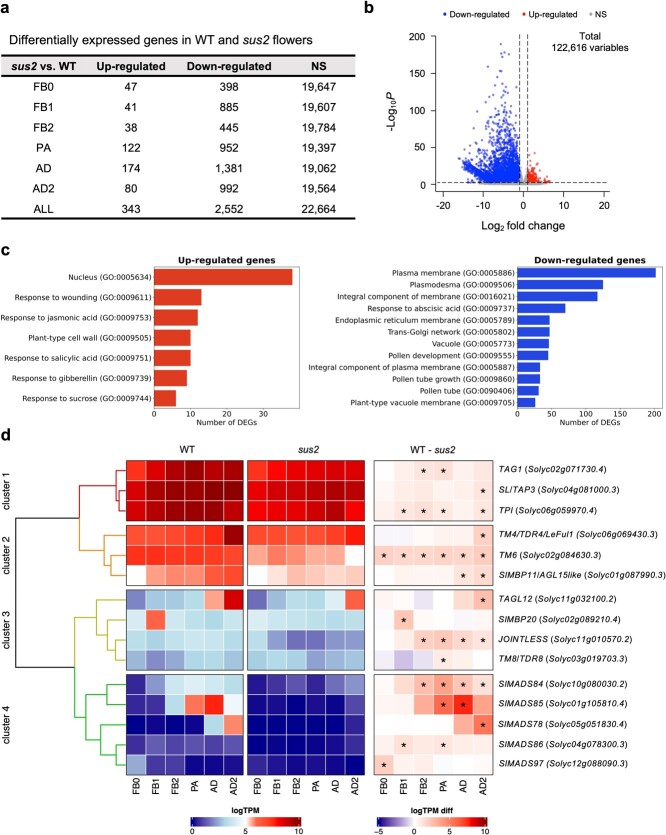
Transcriptomic changes in *sus2* mutant flowers. (a) A total of 2895 DEGs were detected across all analyzed flower developmental stages. (b) Most of these DEGs were found downregulated in *sus2* mutant flowers. (c) Significantly enriched GO terms analysis of down- and upregulated DEGs. (d) Hierarchical clustering of MADS-box genes differentially expressed among WT and *sus2* mutant flowers. The heatmap displays the expression values normalized as the binary logarithm of TPM (WT and *sus2* columns), along with the difference between them (WT—*sus2* column) for 15 MADS-box genes in WT and *sus2* across the six flower stages analyzed. The asterisk indicates differential expression with a false discovery rate-adjusted *P* < 0.01, as determined by the Wald test in the DEseq2 package. FB0, flower bud 0; FB1, flower bud 1; FB2, flower bud 2; PA, flowers at pre-anthesis stage; AD, flowers at anthesis day stage; and AD2, flowers 2 days past anthesis

The results obtained showed a total number of 2895 differentially expressed genes (DEGs) between WT and *sus2* mutant plants across all floral developmental stages, with 343 DEGs upregulated and 2552 downregulated (Fig. 5a and b; Supplementary Tables S1 and S2). Functional relevance of the detected DEGs was performed by Gene Ontology (GO) and Kyoto Encyclopedia of Genes and Genomes (KEGG) pathway enrichment analysis. The most remarkably GO terms among downregulated DEGs included those related to pollen development, pollen tube growth, pollen exine formation, and anther wall tapetum formation, whereas GO terms related with nucleus and plant hormone synthesis such as gibberellins, salicylic, and jasmonic acid were found for the significantly upregulated DEGs (Fig. 5c; Supplementary Table S3). Regarding the KEGG enrichment analysis, upregulated DEGs were enriched in pathways related with biosynthesis of amino acids and secondary metabolites, and plant hormone signal transduction, while downregulated DEGs were enriched in sugar interconversion pathways (Supplementary Table S4).

A detailed analysis of the DEGs revealed a significant downregulation of the *TM6* gene across all developmental stages, despite that *TM6* transcripts were likewise detected in *sus2* flowers (Fig. 5d). Moreover, significant alterations in the expression levels of other 14 MADS-box genes related to flower development were identified, which were also found to be downregulated (Fig. 5d). Four clusters were distinguished based on gene expression abundance. Clusters 1 and 2 comprise genes that were strongly expressed in both WT and mutant plants across flower development, whereas clusters 3 and 4 include genes with lower expression levels in these floral tissues (Fig. 5d). The *TM6* paralogous *SL/TAP3* was found to be significantly downregulated at the late AD2 flower stage. Regarding the rest of B-class function genes, no alterations were observed in the expression level of *TPIB*, although *TPI* downregulation was significant in four flower developmental stages. As for the remaining differentially expressed MADS-box genes, different expression dynamics were observed for the C-class genes *TOMATO AGAMOUS 1* (*TAG1*) and *ARLEQUIN/TAG-LIKE 1 (ALQ/TAGL1*). Downregulation of *TAG1* was evident in FB2 and PA stages (Fig. 5d), while no significant changes were observed in the transcript levels of *ALQ/TAGL1*. In addition, we found *JOINTLESS*, a MADS-box gene essential for the fruit abscission zone development, to be significantly downregulated in *sus2* mutant flowers from FB2 to AD2. During these same stages, the *SlMADS84* gene showed differential expression (Fig. 5d). Another nine MADS-box genes were also found to be differentially expressed in *sus2* flowers, such as *TAGL12* (Supplementary Tables S1 and S2).

Given that *sus2* develops carpelloid stamens and did not produce pollen, we examined in detail the transcriptional activity of pollen development-related genes (Supplementary Fig. S3). One hundred ten genes were found to be differentially expressed in at least one of the analyzed floral developmental stages. Ninety-four percent of these genes were found to be differentially downregulated in the *sus2* mutant. Up to eight clusters were identified based on the expression profiles of pollen development-related genes in WT and mutant plants (Supplementary Fig. S3; Supplementary Table S5). It is noteworthy that genes in cluster 1 were strongly downregulated from FB1 to AD2. Among these genes, it was evident the significant downregulation of genes related to male gametophyte development such as *LATE ANTHER TOMATO 52* (*LAT52*), involved in pollen germination [17]. Genes in cluster 4 and 6 were found to be differentially expressed in the late stages of flower development (from PA to AD2), while clusters 5, 7, and 8 include genes differentially repressed in *sus2* from FB0 to FB2, which suggests that these genes play a key role in early stages of pollen development. Located in cluster 5 is the *MALE STERILE 10^35^* (*MS10^35^*) gene, whose loss of function is related to defective meiosis and tapetum malformation during anthers development [18]. Within the cluster 8, *SlAMS* was found, the tomato homologue of the Arabidopsis *ABORTED MICROSPORES* (*AMS*), which is involved in early tapetum development and its degradation during later stages of flower development [19]. Finally, no changes were detected in the expression of the tomato *MEDIATOR SUBUNIT18* (*SlMED18*) gene, whose loss-of-function mutant shows a fruit phenotype and stamens to carpels homeotic conversion similar to *sus2*, albeit with incomplete penetrance of less than 10% of its fruits [20].

Furthermore, the loss of function of *TM6* significantly increases the expression of carpel specific genes (Supplementary Table S1). This is the case of the *SlTTS* gene, a pistil specific gene orthologue from the tobacco *TRANSMITTING TISSUE SPECIFIC* gene [21], which was found to be upregulated in *sus2* flowers throughout all the developmental stages analyzed. The upregulation of the tomato *CRABS CLAW b* (*SlCRCb*) paralogue at FB1, PA, and AD stages—a gene that plays a key role in carpel formation and flower meristem determinacy [22]—is also noteworthy.

**Figure 6:**
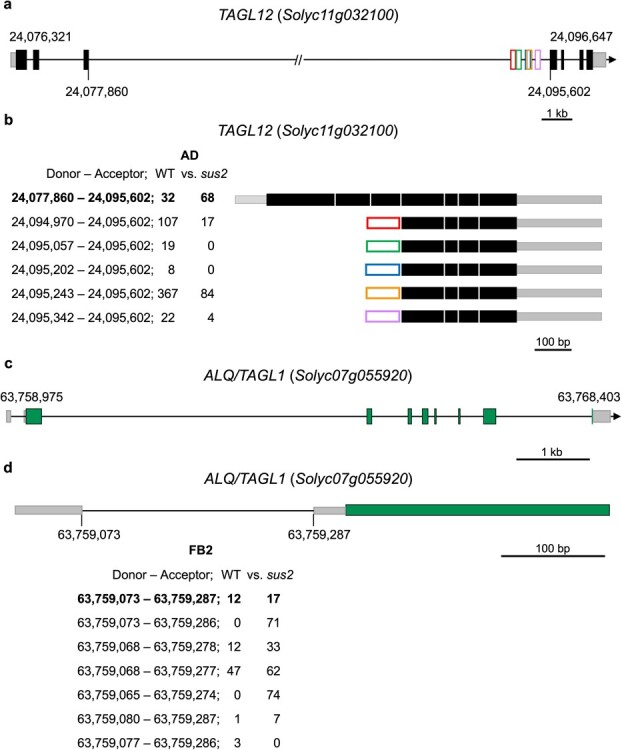
mRNA processing alterations caused by the *sus2* mutation in *TAGL12* and *ALQ/TAGL1* genes. (a) Genomic organization of the *TAGL12* gene. The 5′ and 3′ UTR regions have been depicted as gray boxes, and the coding exons of the gene model have been painted as black boxes. Lines between boxes represent introns, and in the case of the third intron this has been artificially reduced to fit in. Numbers (bp) in the gene model indicate the start and end of transcription, while the numbers below the gene model mark the position of the third exon where the alternative processing occurs. Exons derived from each AS event have been represented as boxes of different colors. (b) All transcripts detected in the transcriptomic analysis are represented with the position of the donor and acceptor sites used. The number of reads supporting each AS site in WT and *sus2* flowers at the AD stage is presented below. (c) Genomic organization of the *ALQ/TAGL1* gene. The 5′ and 3′ UTR regions are indicated as gray boxes, the coding exons are painted as black boxes, and the lines between boxes represent introns. (d) Alternative processing of the first intron of the *ALQ/TAGL1* gene. The amount of each of the transcripts detected in the WT and *sus2* flowers at FB2 stage is shown

In order to confirm the expression levels detected by RNA-seq, a comparative real-time PCR (RT-PCR) analysis was carried out. Correlation among both detection methods was proved by means of a Spearman test, which was performed on RNA-seq TPM values and ∆CT values (Supplementary Fig. S4). Regarding the B-class genes, the analysis allowed to confirm downregulation of *TM6* in *sus2*, suggesting this gene regulates its own expression, as well as other MADS-box genes in different stages of flower development (Supplementary Fig. S5). On the other hand, minor alterations in the expression level of the *TM6* paralogous *SL/TAP3* were detected at FB0, FB1, and PA stages, whereas more significant differences were accounted for AD2, consistent with the findings from the RNA-seq analysis. Similarly, the expression levels of the *TPI* gene were found to be downregulated in *sus2* at five of the six flower developmental stages evaluated (Supplementary Fig. S5). We also analyzed the expression of the C-class *TAG1* gene, which was found to be downregulated in *sus2* from FB1 to PA and in AD2 (Supplementary Fig. S5). Furthermore, the *JOINTLESS* MADS-box gene expression was found to be downregulated in *sus2* from FB1 to AD2 (Supplementary Fig. S5), as previously observed in the RNA-seq analysis. The expression levels of genes related to pollen and stigma development was additionally studied. Thus, downregulation of the male gametophyte-specific *MS10^35^*, *SlAMS*, and *LAT52* genes was evident across developmental stages. Conversely, the stigma-specific *SlTTS* gene was highly induced from FB0 to AD stages (Supplementary Fig. S5), consistent with what was observed in the RNA-seq analysis.

### The *sus2* mutation alters the processing of transcripts

To further assess the implication of *TM6* not only in the control of transcription but also in other aspects of gene expression regulation, we conducted a study of alternative processing of genes expressed in the different developmental stages of floral buds, which was focused on local splicing variation (LSV) analysis. The mRNA variant reads were compared among WT and *sus2* mutant samples using a marginal percent selected index (PSI), which allowed us to account the ratio of reads mapping to every gene and supporting each splicing event. The splicing variants detected for both genotypes in each flower developmental stage analyzed are showed in Supplementary Table S6, which includes gene name, chromosomic location, and function description, as well as canonical splicing sites differential use (SS with LSV) and alternative splicing (AS) events information, i.e. 5′ donor or 3′ acceptor splicing sites (A5SS/A3SS), exon skipping (ES) or intron retention (IR). Delta PSI values for each detected isoform, as well as gene transcriptional status as assessed by means of RNA-seq analysis are also shown in Supplementary Table S6.

A total number of 2384 AS isoforms from 1278 genes were detected using this analysis, from which 595 hold a donor A5SS site (24.95%), 665 had an acceptor A3SS site (27.89%), 783 displayed IR (32.84%), and 341 exhibited ES (14.30%). Different types of transcription factors were found to experience AS between both genotypes. Of particularly note is the case of MADS-box genes related to flower development for which differential use of splicing sites was found between both genotypes. Such is the case of *TAGL12* (*Solyc11g032100*), which has been found to be mainly expressed in flowers, and the *ALQ/TAGL1* (*Solyc07g055920*) gene, universally expressed during flower development (Supplementary Fig. S6).

At the flower stage of AD, the *TAGL12* gene experiences alternative transcription initiation located in the third intron. Remarkably, all the alternative isoforms detected are more frequently found in WT flowers than in *sus2* flowers (Fig. 6a and b). Moreover, alternative isoforms of the *ALQ/TAGL1* (*Solyc07g055920*) gene were also detected from the FB0 to PA flower stages (Fig. 6c). Interestingly, all the wide range of isoforms detected for this gene have in common the presence of four alternative donor 5′ and four acceptor 3′ splicing sites between the first and second exons, as shown as an example in Fig. 6d for the FB2 flower stage.

## Discussion

### Transcriptional activity of *TM6* is essential for flower identity maintenance

Over the past decades, great progress has been made in understanding the genetic network that controls flower development, much of which has been based on the studies performed in the model species *A. thaliana* and *A. majus*. The genes underlying this crucial developmental process are described in the ABC model [1, 6, 7], which is widely conserved among angiosperms. Extension of this model to other species of agronomic interest, such as tomato, has been possible thanks to the characterization of homeotic mutants, most of them of spontaneous origin. This approach, combined with the completion of the tomato genome sequencing, has allowed to describe the genes accounting for each of the ABCDE gene functions, proving that the establishment of floral organs is controlled by highly conserved molecular mechanisms. However, for some of the genes predicted in the model, no tomato mutants have been reported. This absence of mutants hinders our ability to uncover genetic variability that underlies the diversity of reproductive developmental patterns in this crop species. Here we report a tomato mutant identified in a chemically mutagenized population, which is characterized by aberrant petal and stamen morphology, as well as male sterility. Through mapping-by-sequencing and co-segregation test, we have confirmed that a single nucleotide deletion in the MADS-box gene *TM6* is responsible for this mutant phenotype.

The *TM6* gene belongs to a paralogous lineage of *SL/TAP3* arisen from of a duplication event, and it has been proposed to be involved in stamen development [13]. Differential expression patterns have been accounted for both genes and it has been assumed that this may, to some extent, explain their partially divergent functions, given that *SL/TAP3* expression is restricted to petals and stamens of young developing flowers, whereas *TM6* transcripts can be detected in petals, stamens, and carpel primordia of young flowers [14]. Tomato mutants affecting *TM6* gene had not been reported so far; however, de Martino *et al.* [13] performed *TM6* functional characterization by means of RNAi silencing, which resulted in aberrant stamen development but no homeotic changes in petals. These authors, however, reported homeotic transformations in the *sl/tap3* mutant affecting petals and stamens, which raises controversy about the role of *TM6* in petal development. Nevertheless, the absence of petal identity changes observed by de Martino *et al.* [13] in petals of *TM6* RNAi silencing lines may be attributed to the possibility that these lines retained some residual expression levels of this gene, which could have been sufficient for the development of petal primordia. Furthermore, constitutive expression of both *SL/TAP3* and *TM6* under the control of a strong 35S promoter has proved to rescue the *sl/tap3* mutant phenotype in a similar degree [13]. All of these previous findings, together with the evidence provided by the *sus2* mutant phenotype, outlines the implication of *TM6* in the second whorl development and proves its nature as a B-class gene.

The *AP3*/*TM6* lineages have been widely studied in other Solanaceae species such as *Petunia hybrida*, where the downregulation of *AP3* orthologue *DEF* causes homeotic changes, transforming petals into sepals [23]. Petunia *PhTM6* has a similar expression pattern to that of tomato *TM6,* since its transcripts can be detected in stamens and carpels of young floral buds, and this expression is maintained in the fourth whorl during later stages of flower development, in a remarkably similar way to C-class genes [24]. In fact, although *PhTM6* has not been related with petal formation, petal defects observed in the *def* mutant can be restored by complementation with an overexpression construct of *PhTM6*, which confirms that both genes act redundantly in regulating petals and stamen identity [25].

Recently, *TM6* has been described as the candidate gene for the *male sterile-15* (*ms15*) locus, since *ms15* mutants bear mutated alleles of *TM6* [26]. The *ms15* mutant plants develop flowers with reduced and deformed anthers and exerted stigmas, traits that facilitate hand pollination. This is the ultimate usefulness of this kind of mutants for hybrid seed production in crop species like tomato [27]. Nevertheless, unlike what has been observed in the *sus2* mutant, there have been no reported changes in petal morphology in *ms15* mutant plants, suggesting that different domains of the TM6 protein are required to develop specific floral phenotypic traits. Taken together, all these results shed light on the role of *TM6* in determining petal and stamen identity in a higher eudicot species like tomato, and provide new evidence about the functional divergence hypothesis previously assumed for the two *AP3* paralogous lineages [13]. Furthermore, our results demonstrate that the loss-of-function allele of *TM6* identified in the *sus2* mutant leads to changes in cell identity in the second and third whorl organs. Therefore, *TM6* can be considered as a true homeotic B-class gene.

### *TM6* loss of function impairs transcriptional activity of MADS-box genes

An RNA-seq transcriptomic analysis was performed to identify putative targets of *TM6* whose expression was altered by the loss of function of this gene. GO enrichment analysis concluded that among the functionally enriched categories were those related to pollen and stamen development, since numerous genes related to both processes were found to be severely downregulated. These results agree with the lack of pollen grain formation reported both in *sus2* (Fig. 1c) and *ms15* [26] mutant flowers, both bearing mutated alleles of *TM6*. Similar observations were also reported in previous studies carried out in the model species *A. thaliana* [28]. Together, this evidence reinforces the *TM6* function in the control of the male gametophyte formation.

Transcriptomic findings revealed that, despite the downregulation of *TM6* in *sus2* flowers, it is expressed in all assessed floral stages. This downregulation may be attributed to the self-regulation of *TM6* expression. Nevertheless, it cannot be ruled out that *TM6* transcripts were targeted for the nonsense-mediated mRNA decay pathway [29] as the *sus2* mutation leads to the generation of a translation termination codon that is positioned in an abnormal context. Moreover, a detailed analysis of MADS-box genes related to flower development allowed us to elucidate the different expression dynamics resulting from the *TM6* loss of function. Particularly noteworthy is the relation among the paralogous *TAP3*/*TM6* and the other clade of B-class genes *TPI/TPIB.* Transcriptomic analysis suggests that *SL/TAP3* expression remains almost unaltered until the late flower developmental stage of AD2. Furthermore, while no changes were observed for *TPIB* relative expression, downregulation of *TPI* was evident from FB0 to PA stages, as well as in the late AD2 stage, in a similar way to the observed in petals and stamens of *ms15* mutant plants [26]. Previous studies have demonstrated that *SL/TAP3* loss of function does not alter the expression of *TM6* or *TPI* [13, 30], thus providing strong evidence for the assumption that no changes in B-class gene expression is expected from the downregulation of their paralogous genes. Moreover, Guo *et al.* [12] have described that RNAi silencing lines of the *TPIB* gene do not show alterations in the expression profile of the paralogous *TPI*, although they do show strong induction of *TM6*. Our findings suggest that, while *SL/TAP3* expression mainly changes in AD2 mutant flowers, the loss of function of its paralogous *TM6* results in *TPI* downregulation in almost all the evaluated flower development stages. Thus, a strong relationship may be assumed between both B-class genes clades *DEF*/*AP3* and *GLO*/*PI*. In fact, cross-regulation between the *PI* and eu*AP3* genes to maintain their continued expression has been observed in Arabidopsis and Antirrhinum [31]. Since all these observations suggest that downregulation of *TM6* modifies mainly the expression of the tomato *PI* orthologues, further experiments should be achieved focused on B-class genes interaction in different loss-of-function mutants.

Regarding the expression of C-class genes, *TAG1* was found to be repressed in FB2 and PA stages, while *ALQ/TAGL1* experienced no changes. Both C-class genes, *TAG1* and *ALQ/TAGL1*, belong to the euAG and PLE lineages that arose from duplication of the tomato *AG* clade [32]. Both genes are reported to have similar expression patterns during flower development, as their transcripts preferably accumulate in stamens and carpels [33, 34]. In the case of the *sus2* mutant flowers, downregulation was observed only for *TAG1*, which indicates functional divergence among both genes regarding the implication of TM6 in their regulation. Moreover, whereas *TAG1* is involved in stamen and carpel identity specification [33], *ALQ/TAGL1* function is not restricted to flower development but it extends to fruit ripening [35]. Nevertheless, some partial functional redundancy concerning floral whorl identity is also evident, since *ALQ/TAGL1* overexpression lines exhibit homeotic changes from sepals to carpels and from petals to stamens [34], in a similar way to *TAG1* overexpression lines [33]. Given the effect of *TM6* loss-of-function on expression levels of the *TAG1* C-class gene, as well as the *SL/TAP3* and *TPI* B-class genes, it could be assumed that *TM6* plays a crucial role in maintaining the balance between different homeotic gene functions.

Finally, the *SlCRCb* gene, which is related to carpel development, was found to be upregulated, resembling the observed induction of the *CRC* gene in response to *AP3* loss of function in the model species *Arabidopsis thaliana* [28]. Castañeda *et al.* [22] have described that both tomato CRC paralogues (*SlCRCa* and *SlCRCb*) function in a redundant manner acting as positive regulators of floral determinacy, given they maintain normal determination of the floral meristem and carpel development. The antagonistic relationship between *CRC* and B-class genes in carpel development has been proved in Arabidopsis, where *CRC* has been found to be ectopically expressed in the third whorl of null *ap3* mutants, resulting in carpel development due to homeotic changes in stamens [36]. Thus, our results contribute to provide new evidence of the antagonistic relationship between both B- and C-class genes in tomato.

### *TM6* maintains the balance of AS of MADS-box genes involved in flower development

Although transcriptional alterations occasioned by homeotic mutants have received great attention, the control of gene expression includes other less-studied events such as AS, which enable a gene to encode multiple transcripts and potentially different proteins. Indeed, this work represents the first report on the study of differential mRNA processing promoted by the loss of function of a floral homeotic gene, despite the role of AS in generating phenotypic diversity, which arises not only by the different protein isoforms, but also by regulating transcript stability or altering the balance between functional and nonfunctional transcripts [37]. Up to seven events have been described that cause AS including alternative transcription start and termination sites [38]. Among these events, IR has been the most frequently detected in our transcriptomic analysis, which appears in the 32.84% of the AS variants, confirming that IR is the most common form of AS in plants [39].

Taking into account the different mechanisms by which AS is generated [37], it could be hypothesized that the loss of function of the TM6 MADS-box protein generated from the *sus2* allele leads to changes in the levels of certain components of the spliceosome. These changes may result in alternative processing of both the 5′ donor and 3′ acceptor sites, and when both events coincide, they lead to IR. However, in the case of the *ALQ/TAGL1* gene, the AS events that we have observed involved new 5′ donor and 3′ acceptor sites between the first and second exons of the gene, but none of these events cause IR, which demonstrates the precision of the spliceosome. Although no changes occur in the open reading frame of these *ALQ/TAGL1* alternative isoforms, it is remarkable that canonical acceptor and donor splice sites are not present in any of them, even in the reference annotated gene model, which suggests that this splice site may be recognized with limited accuracy by the cellular machinery. In the case of the *TAGL12* gene, the AS events we have observed include new transcription start sites located within the third and larger intron of the gene, which give rise to up five new transcripts detected in WT flowers.

MADS-box proteins show high specificity in controlling the transcription of their targets. This specificity is achieved through a combination of factors, including protein dimerization via their K boxes, binding of the dimer to its targets through the MADS domains, tetramerization of the protein complex, and a preference for certain distances between the targets of the formed tetramer [40]. It is plausible to hypothesize that TM6 is directly involved in the transcription of the AS variants detected in our analysis. This hypothesis is supported by the shift from higher frequency of AS variants in WT flowers to very low occurrence in *sus2* mutant flowers. Although the functionality of the new variants of the *TALG12* gene has not been demonstrated, they are the most abundant transcripts of the gene at certain stages of development (Fig. 6). Remarkably, all AS variants of the *TAGL12* gene have the potential to encode putative proteins that lack the MADS-box but retain a significant portion of the K-box (Supplementary Fig. S7). Whether these truncated forms of TAGL12 are translated and have any functional capacity or whether the AS variants only participate in the balance and stability of the *TAGL12* transcripts remains to be determined.

AS events in members of the MADS-box gene family has been previously reported [41]. Such is the case of the *A. thaliana* E-class gene *ARABIDOPSIS BSISTER* (*ABS*), which regulates ovule development and seed pigmentation [42]. Two alternative isoforms have been reported for this gene, with one lacking the last five amino acids at the end of the K-box. Although no apparent alterations were reported for protein complex formation, a construct of this isoform under the 35S CAMV promoter fails to restore the *abs* mutant phenotype, proving the direct impact of AS on protein functionality [42]. The effects of AS on the functionality of MADS-domain transcription factors have also been demonstrated through the study of *FLOWERING LOCUS M* (*FLM*), a gene involved in the temperature-dependent regulation of flowering time in Arabidopsis. It has been described that temperature variation leads to the predominant accumulation of specific splicing forms of *FLM* transcripts. Proteins produced from these alternative transcripts interact with SHORT VEGETATIVE PHASE, another MADS-domain protein, resulting in the formation of higher order complexes, which either promote or suppress flowering based on the specific *FLM* isoform incorporated into the dimer [43]. More recently, alternative isoforms of the *ALQ*/*TAGL1* gene have been reported in the background of the tomato *green stripe* (*gs*) mutant. The phenotype of this mutant is due to a hypermethylated allele of *TAGL1*, which alters chloroplast development and carotenoid accumulation [44].

Results reported here contribute to shed light on the regulatory interactions of MADS-box genes in flower development and further strengthen the evidence of the regulatory function of *TM6* during petal and stamen morphogenesis. Although great progress has been achieved in understanding flower development and the involvement of MADS-box transcription factors in this process, future research should focus on protein interactions and Chromatin Immunoprecipitation sequencing (ChIP-seq) analysis to fully dissect the regulatory interactions of *TM6* with other key players in floral organ identity.

## Materials and methods

### Plant material and phenotypic characterization

The *sus2* mutant was identified as part of the screening of a chemically mutagenized population obtained in the tomato cultivar Moneymaker (MM) using EMS, as previously described [15]. The *sus2* mutant was selected based on the conspicuous homeotic changes observed in the flowers of an M_2_ segregating family. Phenotypic characterization was performed in both M_2_ and M_3_ populations, which were grown alongside control MM plants. The wild relative *S. pimpinellifolium* accession LA1589, retrieved from the Tomato Genetics Resource Center (http://tgrc.ucdavis.edu/), was employed to generate the F_2_ mapping population. All experiments were conducted under greenhouse conditions, as previously described by Fonseca *et al.* [15].

### Pollen viability assays

Pollen viability was assessed *in vitro* by staining pollen grains with a 0.5% solution of 2,3,5-triphenil tetrazolium chloride in a 0.5 M solution of sucrose. A total of 20 WT and 20 *sus2* mutant flowers were employed. Incubation took place for two hours at 50°C in darkness in a humid box. Results were visualized using OPTIPHOT-2 (Nikon) optical microscopy.

### Scanning electron microscopy

Epidermal cell morphology of the four floral whorls of both WT and *sus2* mutant plants was assessed by SEM, following the methodology previously described by Lozano *et al.* [14]. Briefly, plant material was fixed in an FAEG solution (3.7% formaldehyde, 5.0% acetic acid, 50% absolute ethanol, and 0.5% glutaraldehyde), and after 72 hours of incubation, it was stored in 70% ethanol. Samples were dehydrated in increasing concentrations of ethanol and then dried with liquid CO_2_ in a Bal-Tech CPD 030 critical drier. Following this, samples were gold coated using a Bal-Tec SCD005 sputter coater and visualized with a Hitachi S-3500N scanning electron microscope.

### Genetic mapping of the *sus2* mutation

To determine the chromosomic location of the *sus2* mutation, an F_2_ interspecific population composed of 129 plants was assessed. These plants were descendants of an F_1_ plant obtained from a cross between a *sus2* mutant plant and a plant from the wild species *S. pimpinellifolium*, accession LA1589. Leaves from F_2_ plants were frozen in liquid nitrogen and grounded using a Retsch MM301 mixer mill shaker. Genomic DNA of individual F_2_ plants was isolated using a DNAzol® Reagent Kit (Invitrogen Life Technologies, USA) following the manufacturer’s instructions. DNA concentration was estimated using a Nanodrop 2000 Spectrophotometer (ThermoFisher Scientific, USA) and by comparison with DNA standard markers after electrophoresis. All 129 F_2_ plants were individually genotyped, and mapping was carried out using codominant markers distributed along the genome [16]. Genetic linkage and distances were determined using JoinMap® 4 software [45]. A 600 kb candidate interval was identified in chromosome 2 using the markers provided in Supplementary Table S7. Fine mapping was completed using a whole-genome sequencing approach following the methodology described by Yuste-Lisbona *et al.* [46].

DNA pools, comprising equimolar amounts of DNA from 25 WT and 19 mutant F_2_ plants with contrasting phenotype, were sequenced using an Illumina HiSeq2000 platform (Illumina, Inc., USA) with 150 bp paired ends. The obtained reads were deposited in the SRA database at the NCBI under BioProject accession number PRJNA1015112 and aligned against the tomato genome reference sequence version 4.0 (ITAG4.0) using Bowtie2 version 2-2.0.0-b5 with default parameters [47]. Duplicated reads were eliminated using Picard 1.65, and indel realignment was performed using GATK 2.2-8 with default settings [48]. VCFtools was used for variant calling [49]. To determine the allele frequency ratio (i.e. non-reference allele counts/total allele counts) for biallelic variants, the reference and non-reference allele counts for each position were obtained from SAMtools 1.2 [50]. Finally, the candidate chromosomal region to harbor the *sus2* mutation was detected by plotting the average allele frequencies determined for each chromosome using a custom script in the R environment for statistical computing [51]. The results obtained from the *sus2* mutant pool were compared with those sequences retrieved from the WT pool. The *TM6* locus was genotyped using a CAPS marker, which amplifies a 539 bp fragment of the *Solyc02g084630* gene (primer sequences in Supplementary Table S7, marker 47.7 Mb). When digested PCR products with BpmI, the PCR amplicon from the mutant allele was cleaved into 270 and 268 bp fragments, whereas the WT allele abolishes the BpmI recognition site.

### RNA isolation and whole transcriptome sequencing

Total RNA was isolated from flowers harvested at six different developmental stages following the description by Mazzucato *et al*. [52]. These stages include flower bud 0 (FB0), flower bud 1 (FB1), flower bud 2 (FB2), flower at pre-anthesis (PA), flower at anthesis day (AD), and flower 2 days after anthesis (AD2). Three biological replicates were collected per stage for both WT and *sus2* mutant flowers. RNA isolation was performed using TRIzol® Reagent (Invitrogen Life Technologies, USA) following the manufacturer’s protocol. DNA contamination was eliminated by treating the samples with a DNA-free™ DNA Removal Kit (Invitrogen Life Technologies, USA). Samples were sequenced using an Illumina HiSeq2000 platform (Illumina, Inc., USA) with 150 bp paired ends. The obtained reads were deposited in the SRA database at the NCBI under BioProject accession number PRJNA1015144. Sequencing reads were mapped to the tomato reference genome (ITAG4.0) using HISAT 2.2.1 [53] with the ‘very-sensitive’ option. Raw read counts were obtained using the featureCounts tool from the Subread suite 2.0.1 [54]. Transcript per million (TPM) normalization was applied to raw read counts [55], and the resulting normalized expression levels were used for clustering genes and samples. The ComplexHeatmap package in R was used for generating heatmap plots and dendrograms [56]. This approach facilitated a detailed visualization and comparison of expression patterns among different samples and genes.

To test for consistency among RNA-seq replicates, the top 5000 genes with the highest cumulative expression across all samples were selected. These genes were used for hierarchical biclustering analysis of replicates based on *Z*-scores of expression values, represented as TPM. The *Z*-score measures the difference (in absolute value and measured as the number of standard deviations) between the normalized expression level for a given gene and sample compared with the mean normalized expression of that same gene across all samples (Supplementary Fig. S8). As a result, replicate R1 of WT FB2, which showed a discordant clustering behavior when compared with the other two biological replicates, was excluded from further analysis. Differential expression analysis was conducted using the Wald test in the DEseq2 package [57]. Genes with a false discovery rate-adjusted *P* < 0.01 were considered as DEGs. GO term and KEGG pathway enrichment analyses were performed using the Cytoscape plug-in ClueGO [58] for each set of upregulated and downregulated genes. The GO term enrichment drew upon the ITAG4.0 database from agriGO v2.0, while the KEGG pathway enrichment utilized ClueGO’s tomato database for KEGG. The ClueGO network specificity ranged from level 3 to level 8, with connectivity based on a kappa score of 0.5. The Benjamini–Hochberg method was applied for *P*-value corrections, and significance was determined at a corrected *P*-value of <0.01 for GO terms and <0.05 for KEGG pathways.

### Quantitative RT-PCR expression analysis

The same WT and mutant samples employed for RNA-seq were analyzed by quantitative RT-PCR. One microgram of RNA was used for cDNA synthesis with M-MuLV Reverse Transcriptase (ThermoFisher Scientific, USA) using a mixture of random hexamer and oligo(dT)_18_ primers. Three biological and two technical replicates were analyzed by quantitative RT-PCR using the 7300 Real-Time PCR System (Applied Biosystems, ThermoFisher Scientific, USA) and SYBR Green PCR Master Mix (Applied Biosystems, ThermoFisher Scientific, USA). The sequences of the gene-specific primer pairs are listed in Supplementary Table S8. The housekeeping gene *UBIQUITIN3* was employed for sample normalization [20], and the Ct calculation method was employed for the quantitation of relative gene expression [59]. Finally, differences in gene expression levels were statistically analyzed using a Student’s *t*-test (*P <* 0.05).

### AS analysis

Given that AS holds a key role in modulating gene function, mRNA splicing events were identified and quantified as LSVs, as previously described by Vaquero-Garcia *et al.* [60]. For this purpose, we used the MAJIQ software (https://majiq.biociphers.org/) to analyze the same alignments of RNA-seq reads that were used for quantifying gene expression. Therefore, a marginal PSI value was obtained for each LSV and sample, with each LSV representing a splicing event (alternative or canonical) and PSI being the ratio of reads mapping to the gene that supports that splicing event. Next, PSI values were compared between the *sus2* mutant plant samples and the WT plant samples in a stage-by-stage fashion. Isoforms were considered if they exhibited at least one LSV with a delta PSI value ≥0.2, calculated within a confidence interval of 0.95. The delta PSI value was determined as the difference (in absolute value) between the average PSI of *sus2* mutant plants and the average PSI of WT plants for a given LSV.

## Acknowledgements

This research was supported by research grants PID2019-110833RB-C31 and P20_00324 funded by the Spanish Ministry of Science and Innovation (MCIN/AEI/10.13039/501100011033) and the Spanish regional government of Junta de Andalucía (2014-2020 FEDER-Andalusia Operational Program), respectively. The authors would like to thank Campus de Excelencia Internacional Agroalimentario (CeiA3) for providing research facilities.

## Author Contributions

R.L. and J.C. conceived and designed the research. R.F., C.C., and R.L. performed research experiments and data analysis. A.O.-A., F.J.Y.-L., and T.A. contributed new reagents or analytical tools. R.F., R.L., F.J.Y.-L., and J.C. wrote and edited the manuscript. All authors read and approved the manuscript.

## Data Availability

The DNA-seq and RNA-seq data from this article can be found at the Sequence Read Archive (SRA; https://www.ncbi.nlm.nih.gov/sra/) under BioProject accession numbers PRJNA1015112 and PRJNA1015144, respectively.

## Conflict of Interests

The authors declare that they have no known competing financial interests or personal relationships that could have appeared to influence the work reported in this paper.

## Supplementary Data

Supplementary data are available at *Horticulture Research* online.

## Supplementary Material

Web_Material_uhae019
