# Supplementary material for: Insights into the functional role of tomato TM6 as a transcriptional regulator of flower development
Source: Hortic Res. 2024 Jan 16;11(3):uhae019. doi: 10.1093/hr/uhae019 (PMC10923641; doi:10.1093/hr/uhae019)
Supplement: Web_Material_uhae019 [file web_material_uhae019.zip › Supplementary Figures S1-S8.pdf]

Insights into the functional role of tomato *TM6* as transcriptional regulator of flower development

Rocío Fonseca* (0000-0002-7651-2366), Carmen Capel* (ORCID: 0000-0002-9574-3303), Ricardo Lebrón (ORCID: 0000-0002-5120-843X), Ana Ortiz-Atienza (ORCID: 0000-0003-1750-4785), Fernando J. Yuste-Lisbona (ORCID: 0000-0001-9222-7293), Trinidad Angosto (ORCID: 0000-0003-0294-9255), Juan Capel[#] (ORCID: 0000-0002-4327-0604), Rafael Lozano[#] (ORCID: 0000-0001-5458-2075).

Centro de Investigación en Agrosistemas Intensivos Mediterráneos y Biotecnología Agroalimentaria (CIAIMBITAL), Universidad de Almería, Edif. CITE II-B, Carretera de Sacramento s/n, 04120-Almería, Spain.

* R. Fonseca and C. Capel contributed equally to this work.

[#]Corresponding authors

Prof. Juan Capel
Departamento de Biología y Geología (Genética).
Edificio CITE II-B, Universidad de Almería.
Carretera de Sacramento s/n, 04120 Almería, Spain.
Phone: +34 950015889. Fax: +34 950015476.
Email: jcapel@ual.es

Prof. Rafael Lozano
Departamento de Biología y Geología (Genética).
Edificio CITE II-B, Universidad de Almería.
Carretera de Sacramento s/n, 04120 Almería, Spain.
Phone: +34 950015111. Fax: +34 950015476.
Email: rlozano@ual.es

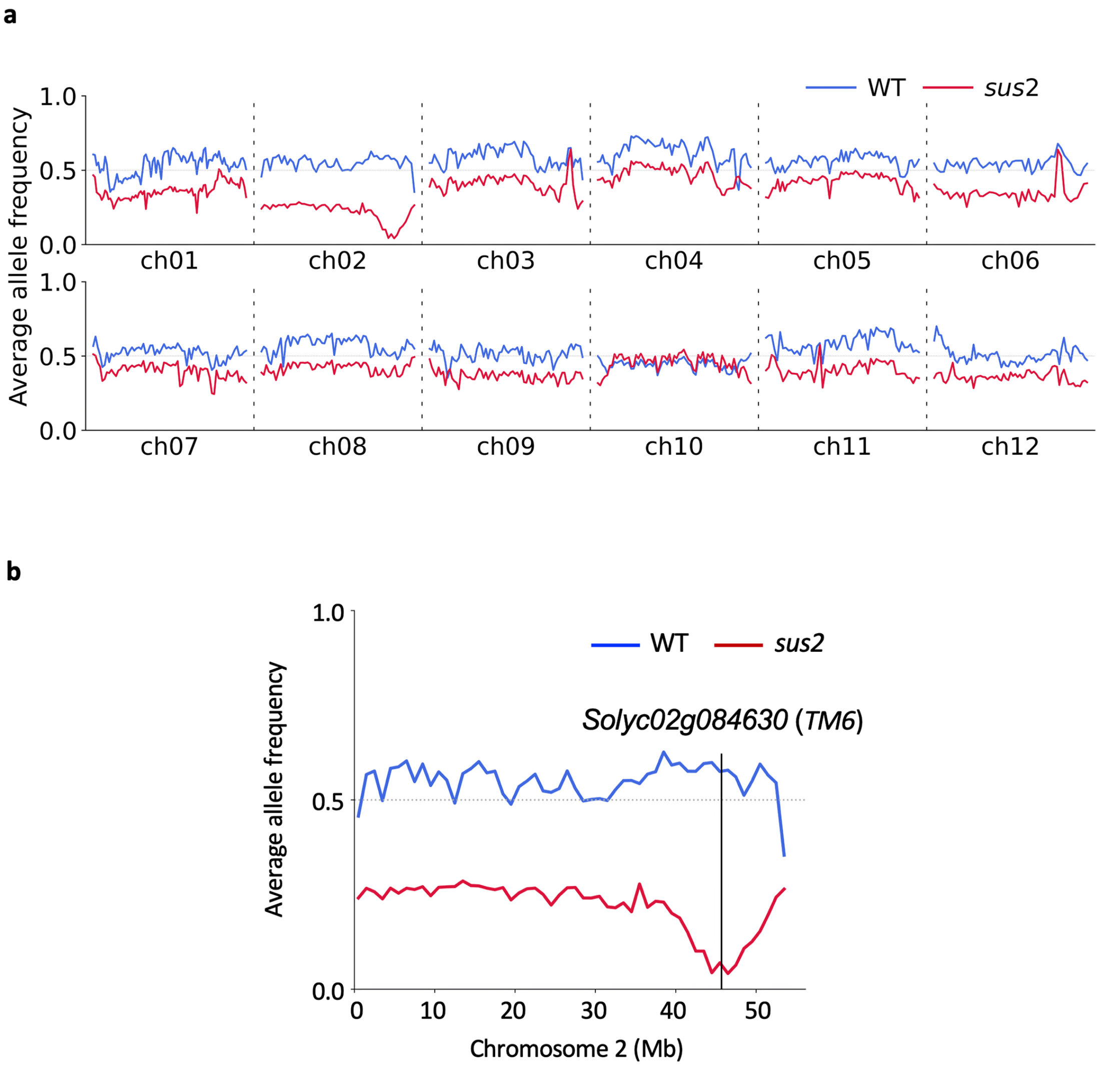

Supplementary Fig. S1. Mapping-by-sequencing identification of the *sus2* mutation. **a** Comparison of the allele frequencies obtained from the sequencing of a DNA pool formed by 25 wild-type (WT) plants and a pool of 19 mutant (*sus2*) plants, all from a F2 population derived from the cross between a *sus2* mutant plant and a plant of the wild relative species *S. pimpinellifolium* accession LA1589 (for details, see Yuste-Lisbona et al. 2021). **b** The distal region of chromosome 2 showed a drop in the average allele frequency indicating that the *sus2* mutation is located in this portion of the chromosome, where a mutation on the *Solyc02g084630* gene syn*. TM6* was identified (vertical line).

```
TM6         MGRGKIEIKKIENSTNRQVTYSKRRNGIFKKAKELTVLCDAKISLIMLSSTRKYHEYTSP   60
sus2        MGRGKIEIKKIENSTNRQVTYSREETVFSRKLKNLLFF----VTLRSLSSC-----YQAP   51
            **********************:......:*.*.*...    ::*..***:     *.:*

TM6         N---TTTKKMIDQYQSALGVDIWSIHYEKMQENLKRLKEINNKLRREIRQRTGEDMSGLN  117
sus2        GSIMSTQAQTLRQKR                                                66
            .   :*..:.:.*.:

TM6         LQELCHLQENITESVAEIRERKYHVIKNQTDTCKKKARNLEEQNGNLVLDLEAKCEDPKY  177

TM6         GVVGVVENEGHYHSAVAFANGVHNLYAFRLQPLHPNLQNEGGFGSRDLRLS          225
```

Supplementary Fig. S2. Alignment of the sequences of the TM6 wild-type protein and the protein coded by the *sus2* mutant allele. Highlighted in yellow are the residues that compose the MADS-box and highlighted in green are the residues that compose the K-box, according to the PROSITE prediction tool. Asterisks (*) mark the positions which have a conserved residue, colons (:) indicate conservation between groups of strongly similar properties scoring > 0.5 in the Gonnet PAM 250 matrix, whereas periods (.) denote conservation between groups of weakly similar properties scoring =< 0.5 in the Gonnet PAM 250 matrix.

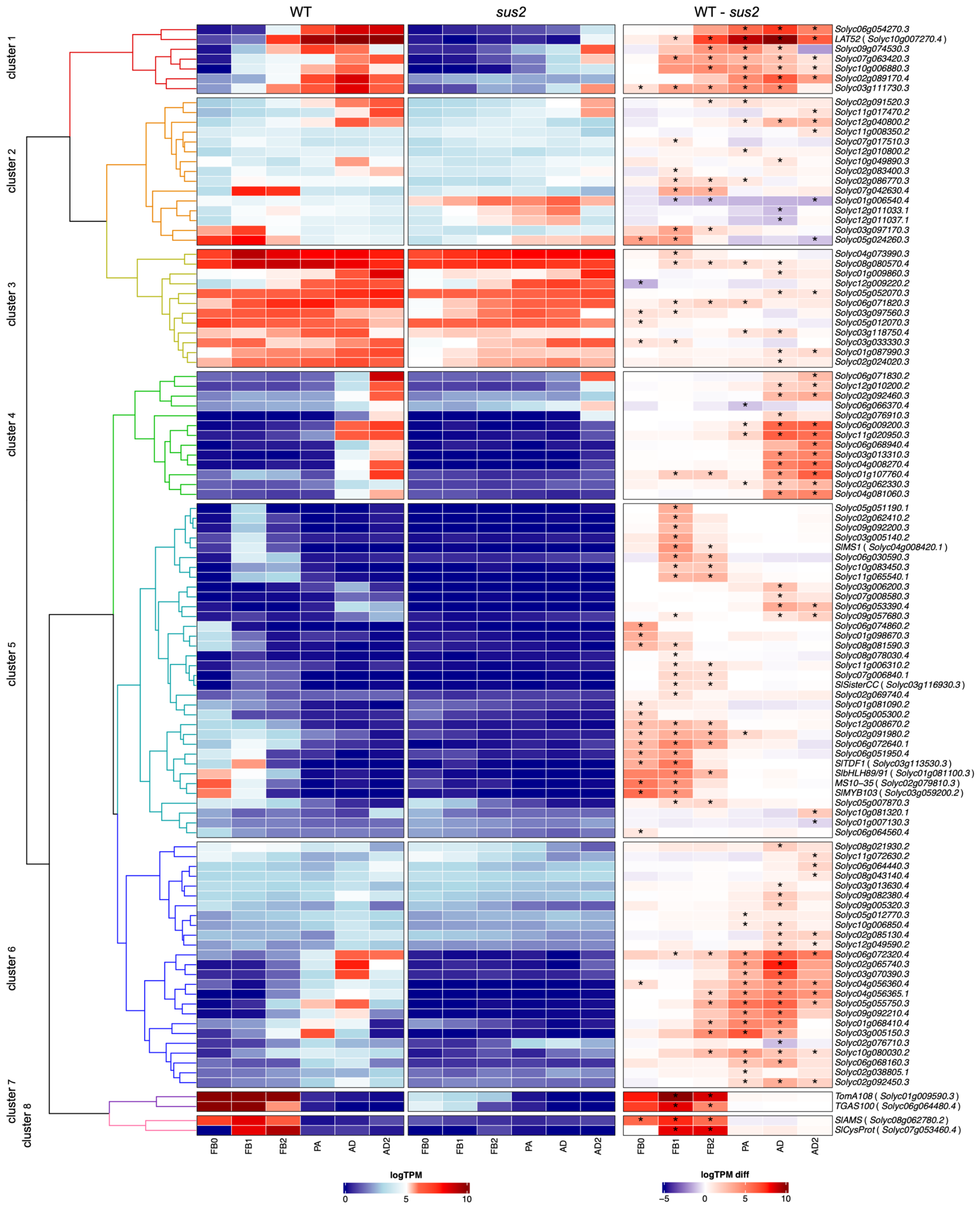

Supplementary Fig. S3. Hierarchical clustering of pollen development-related genes (GO:0009555) differentially expressed among wild-type (WT) and *sus2* mutant flowers. The heatmap displays the expression values normalized as the binary logarithm of Transcript Per Million (TPM), along with the difference between them, in WT and *sus2* across the six flower stages analyzed. The asterisk indicates differential expression with a false discovery rate-adjusted P-value < 0.01, as determined by the Wald test in the DEseq2 package. FB0, flower bud 0; FB1, flower bud 1; FB2, flower bud 2; PA, flowers at pre-anthesis stage; AD, flowers at anthesis day stage; and AD2, flowers two days past anthesis.

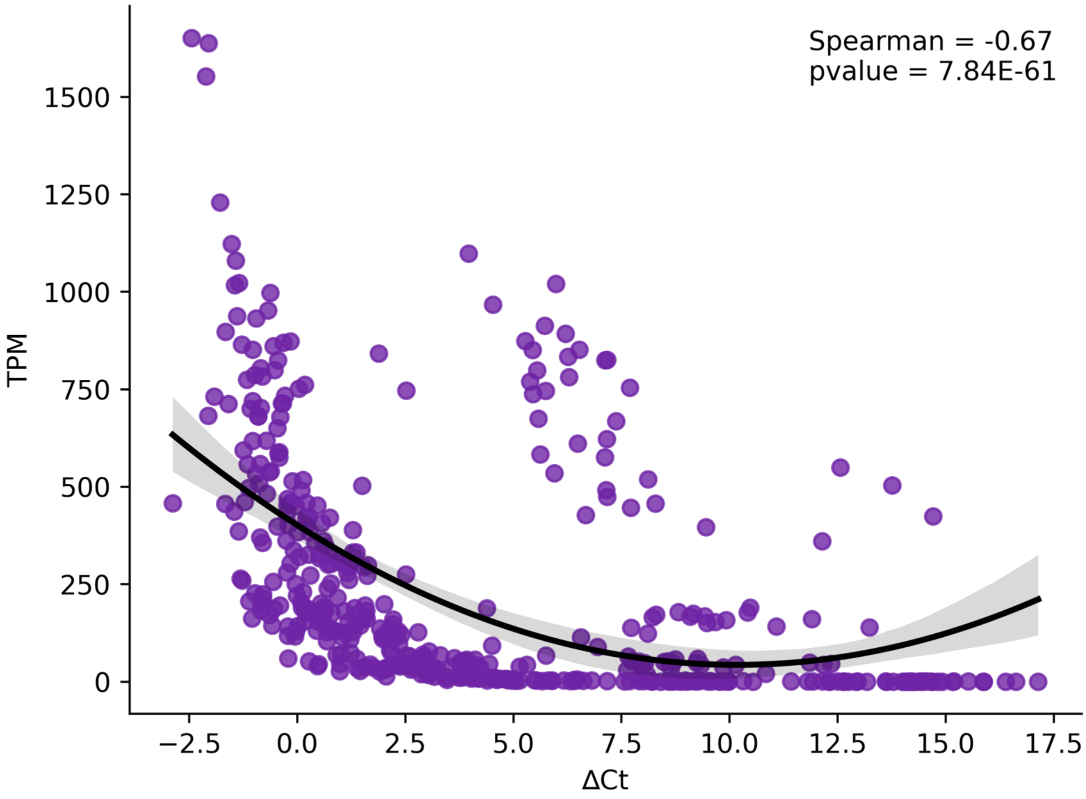

Supplementary Fig. S4. Statistical correlation between RNA-seq and quantitative RT-PCR methods. Correlation was assessed by means of a Spearman test using the Transcript Per Million (TPM) and ∆CT values detected for nine genes in the six flower stages analyzed of wild-type (WT) and *sus2* plants. The correlation analysis includes the three biological replicates of each flower stage and the two technical replicates of the quantitative RT-PCR. Black line indicates the trend of the quadratic regression and grey bands account for the confidence interval (95%) of the quadratic regression.

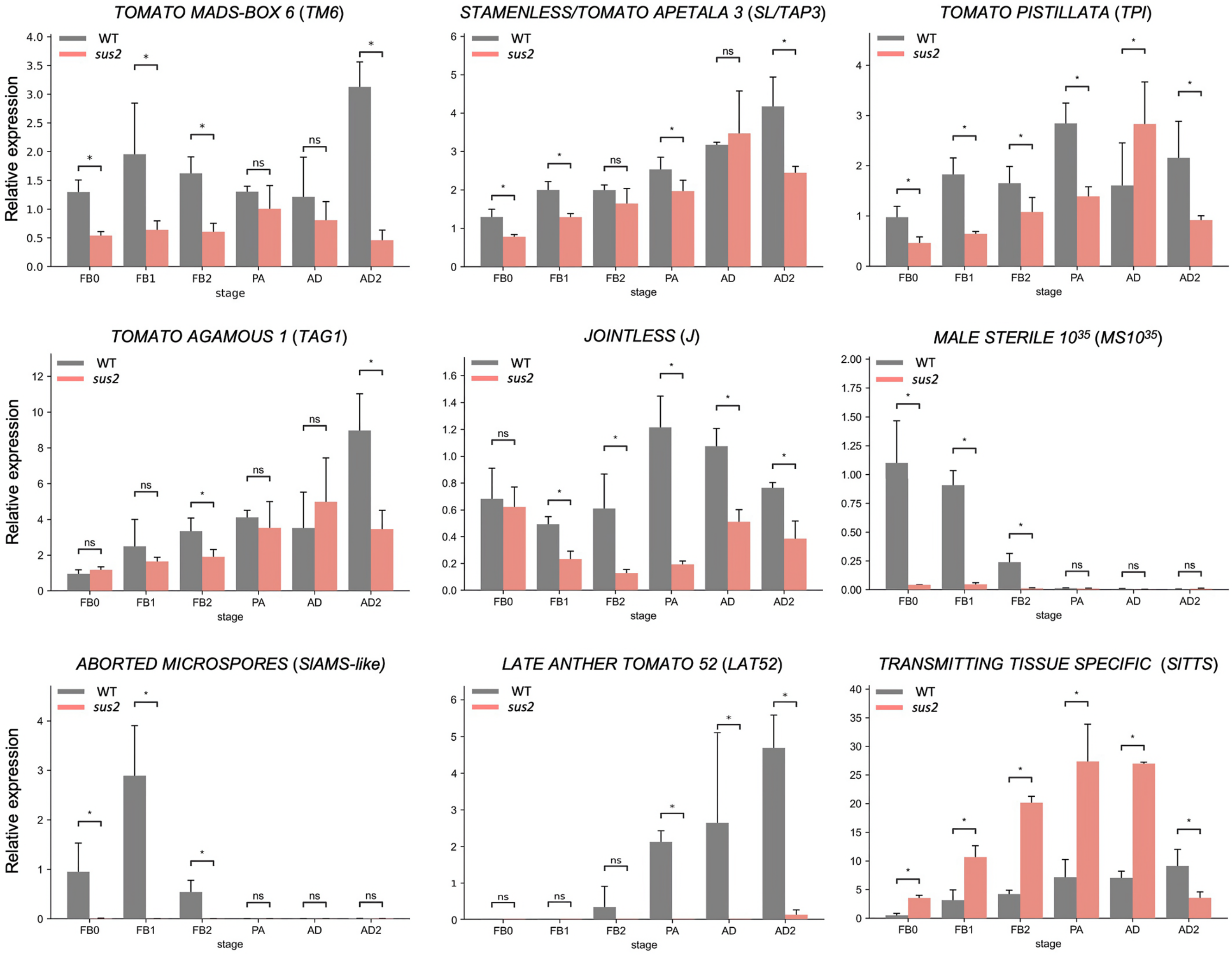

Supplementary Fig. S5. Relative expression of floral identity and pollen development genes assessed by qRT-PCR. Six developmental stages have been analyzed in three biological replicates of wild-type (WT) and *sus2* mutant flowers, i.e. flower buds 0 (FB0), flower buds 1 (FB1), flower buds 2 (FB2), pre- anthesis flower (PA), anthesis day flower (AD) and flowers two days after anthesis (AD2). Asterisk denotes significant differences (Student's t-test, $P < 0.05$); ns, no statistically significant differences.

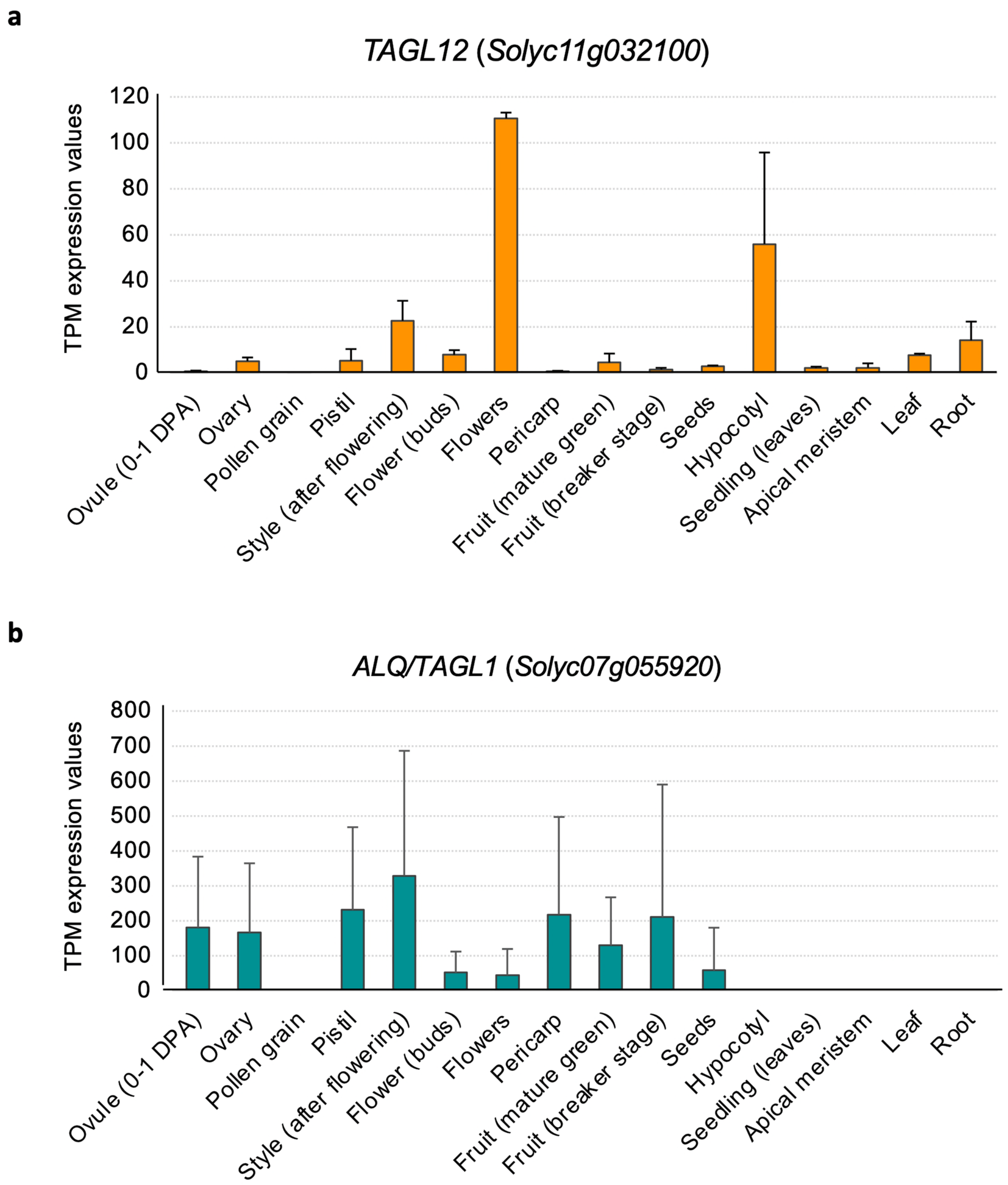

Supplementary Fig. S6. Expression profiles of MADS-box genes found to experience alternative splicing. The *TAGL12* gene was found to be mostly expressed in flowers (**a**), whereas the *ALQ/TAGL1* gene shows a more ubiquitous expression pattern (**b**). Data retrieved from the Evorepro database (https://evorepro.sbs.ntu.edu.sg/).

```
TAGL12        MARGKVQMKRIENPVHRQVTFCKRRAGLLKKAKELSVLCDAEIGLFIFSAHGKLYELATK  60
24.094.970    ------------------------------------------------------------   0
24.095.057    ------------------------------------------------------------   0
24.095.202    ------------------------------------------------------------   0
24.095.243    ------------------------------------------------------------   0
24.095.342    ------------------------------------------------------------   0

TAGL12        GSMQGLIERYIKSTKGVEVAEEAKDTQPLDPKEEINMLKNEIDVLQKGLSYMYGGGAGTM 120
24.094.970    ---------------------------------------------------MYGGGAGTM   9
24.095.057    ---------------------------------------------------MYGGGAGTM   9
24.095.202    ---------------------------------------------------MYGGGAGTM   9
24.095.243    -----------------------------------------MKFQNSRDSYMYGGGAGTM  19
24.095.342    ---------------------------------MSRSLPKIDTYITVPICYMYGGGAGTM  27
                                                                 *********

TAGL12        TLDELHSLEKYLEIWMYHIRSAKMDIMFQEIQLLKNKEGILEAANKYLQDKIDEQYTVTN 180
24.094.970    TLDELHSLEKYLEIWMYHIRSAKMDIMFQEIQLLKNKEGILEAANKYLQDKIDEQYTVTN  69
24.095.057    TLDELHSLEKYLEIWMYHIRSAKMDIMFQEIQLLKNKEGILEAANKYLQDKIDEQYTVTN  69
24.095.202    TLDELHSLEKYLEIWMYHIRSAKMDIMFQEIQLLKNKEGILEAANKYLQDKIDEQYTVTN  69
24.095.243    TLDELHSLEKYLEIWMYHIRSAKMDIMFQEIQLLKNKEGILEAANKYLQDKIDEQYTVTN  79
24.095.342    TLDELHSLEKYLEIWMYHIRSAKMDIMFQEIQLLKNKEGILEAANKYLQDKIDEQYTVTN  87
              ************************************************************

TAGL12        MTQNLTDFQCPLTVQNEIFQF                                        201
24.094.970    MTQNLTDFQCPLTVQNEIFQF                                         90
24.095.057    MTQNLTDFQCPLTVQNEIFQF                                         90
24.095.202    MTQNLTDFQCPLTVQNEIFQF                                         90
24.095.243    MTQNLTDFQCPLTVQNEIFQF                                        100
24.095.342    MTQNLTDFQCPLTVQNEIFQF                                        108
              *********************
```

Supplementary Fig. S7. Alignment of the sequences of the TAGL12 protein and the proteins potentially coded by the variant transcripts detected. Highlighted in yellow are the residues that compose the MADS-box and highlighted in green are the residues that compose the K-box according to the PROSITE prediction tool.

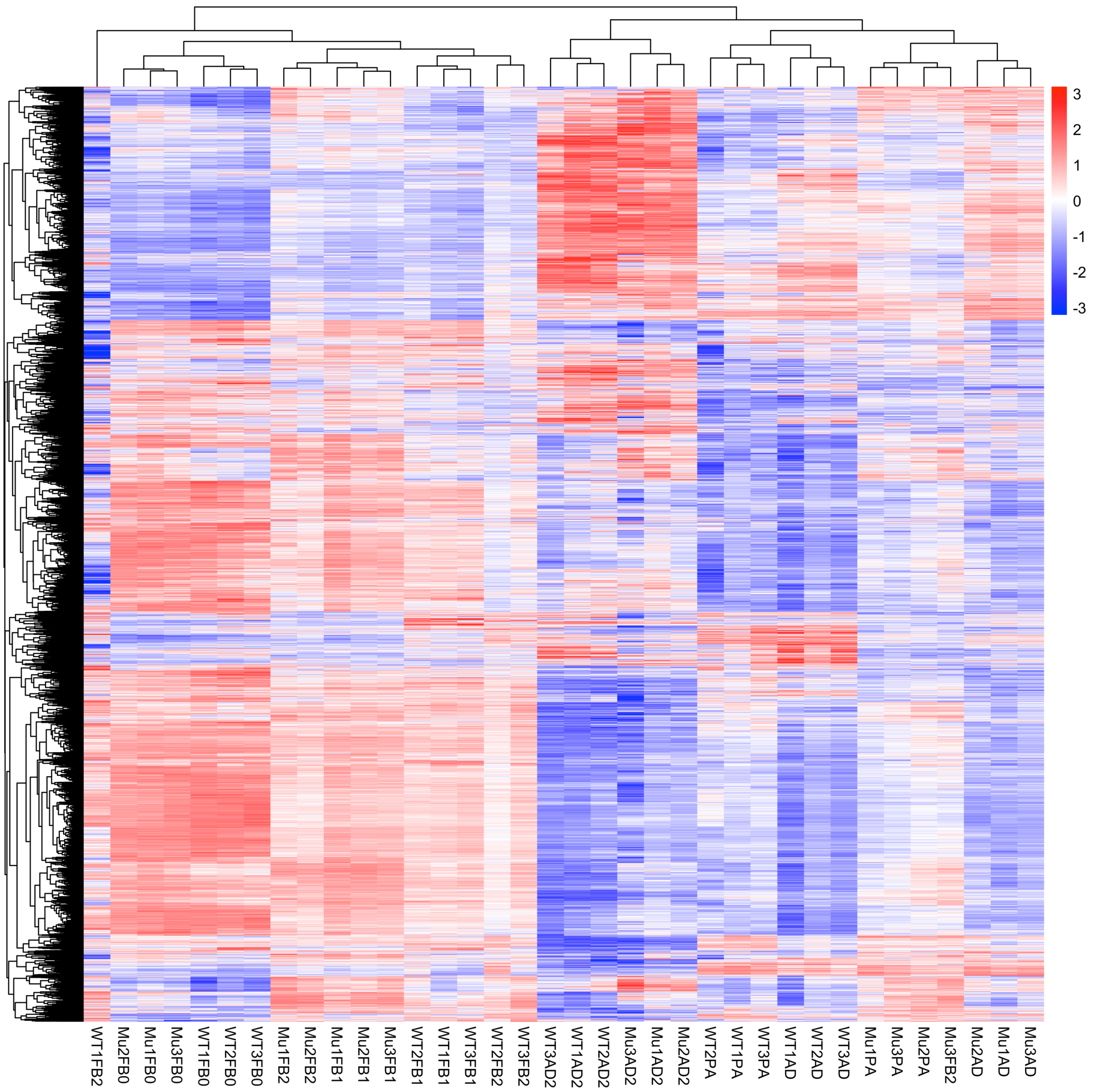

Supplementary Fig. S8. Test for consistency among RNA-seq replicates. The top 5000 genes with the highest cumulative expression across all samples were selected for hierarchical biclustering analysis of replicates based on Z-scores of the expression values of transcripts per million (TPM), with the Z-score being the difference (in absolute value and measured as number of standard deviations), between the normalized expression level for a given gene and a given sample, versus the mean normalized expression of that same gene across all samples. Genotypes: wildtype (WT) and *sus2* mutant (Mu). Developmental stages: flower bud 0 (FB0), flower bud 1 (FB1), flower bud 2 (FB2), flower at pre-anthesis (PA), flower at anthesis day (AD) and flower two days after anthesis (AD2).
